# Invisible exodus: toward a methodology for estimating religious displacement in Nigeria

**DOI:** 10.3389/fsoc.2025.1677251

**Published:** 2025-12-08

**Authors:** Dennis P. Petri, John T. Bainbridge

**Affiliations:** 1 Faculty of Social Sciences, Latin American University of Science and Technology, Barrio Tournón, San José, Costa Rica; 2 International Institute for Religious Freedom, Orlando, FL, United States

**Keywords:** religious displacement, Nigeria, internally displaced persons (IDPs), Christian persecution religious violence, proxy estimation, displacement data gaps, methodology for religious demographics

## Abstract

The purview of the current study is to estimate the religious affiliation of Nigerian displaced persons, specifically to assess the number of internally displaced Christians. Although official data sources such as the Internal Displacement Monitoring Center (IDMC), International Organization for Migration (IOM), and United Nations High Commissioner for Refugees (UNHCR) do not disaggregate internally displaced persons (IDPs) by religion, a growing body of evidence indicates that a substantial proportion of these individuals are Christians displaced as a result of religiously motivated violence. Because major displacement datasets lack religious identifiers, this study develops a proxy-based estimation model that infers the religious composition of displacement through patterns of targeted violence. This approach directly addresses the absence of systematic data on the religious affiliation of victims of forced displacement, offering an innovative method to approximate what existing sources cannot measure. Specifically, we apply state-level ratios of religiously targeted killings from the Observatory for Religious Freedom in Africa (ORFA) to estimate the likely number of displaced Christians in 2023. This study finds that Christian communities in Nigeria experience disproportionate displacement linked to community-targeted violence, with northwestern states Katsina, Sokoto, and Zamfara showing the highest relative disparities and Borno and Taraba contributing the largest absolute numbers. While these patterns align with known hotspots of insecurity, the analysis relies on modeled relationships between killings and displacement and on proxy data, so the findings should be interpreted as indicative rather than definitive. The study underscores the need for more disaggregated humanitarian data to better understand the role of religious persecution in forced migration.

## 1 Introduction

Nigeria, Africa's most populous country, continues to grapple with complex and overlapping crises, including terrorist violence, ethno-religious conflict, and criminal insurgencies, that have triggered one of the continent's most severe internal displacement emergencies (Armed Conflict Location and Event Data Project (ACLED), 2022). As of late 2023, Nigeria had an estimated 3.4 million internally displaced persons (IDPs), including 291,000 new conflict-related displacements that year (Internal Displacement Monitoring Centre (IDMC), 2024). While large in absolute terms, Nigeria's displacement levels remain proportionally lower than in countries such as Sudan, Syria, the Democratic Republic of Congo, Colombia, and Yemen, which collectively accounted for nearly half of the world's conflict-related IDPs in 2023 (United Nations High Commissioner for Refugees (UNHCR), 2024). Nevertheless, the humanitarian and political ramifications are profound, especially given the scale, duration, and religious dynamics of Nigeria's violent conflicts (Expert Group on Refugee and IDP Statistics (EGRIS), 2024; Adeleye and Aremu, 2023; Enobong and Lawanson, 2023; Babajide et al., 2024).

Despite comprehensive tracking by organizations such as the Internal Displacement Monitoring Center (IDMC), the International Organization for Migration (IOM), and the United Nations High Commissioner for Refugees (UNHCR), none of these sources disaggregate IDP data by religious affiliation. This omission obscures key patterns in Nigeria's displacement crisis. As MacLeod (2022) argue, the absence of granular data, especially on identity markers like religion, reflects broader limitations in displacement evidence systems and hampers targeted humanitarian response.

Field reports (Observatory of Religious Freedom in Africa, 2024; Open Doors International, 2025) and qualitative evidence (International Institute for Religious Freedom, 2024) increasingly indicate that Christians, particularly those in the Middle Belt and northern regions, constitute a disproportionately large share of those displaced by violence. This trend appears consistent with the geography of attacks, patterns of religiously motivated community raids, and targeted destruction of Christian institutions. Yet official datasets do not allow for empirical verification of these claims.

Exacerbating this data void, some Christian advocacy groups have made unverified and potentially inflated claims, including the estimate that as many as 5 million Christians have been displaced (see U. S. House Committee on Foreign Affairs, 2023), an assertion made without rigorous data to support it. Such claims often blur the important distinction between “IDPs” and “internal displacements,” two separate metrics tracked by IDMC. IDPs refer to the stock of displaced individuals at the end of the calendar year, each counted once regardless of multiple displacements. By contrast, internal displacements capture the total number of displacement incidents during a year, which may include repeat displacements of the same individuals. Confusing the two can lead to overestimations and policy misdirection.

This paper seeks to clarify and correct the narrative by proposing a transparent methodology for estimating the number of internally displaced Christians in Nigeria. This approach builds on analytical frameworks for engaging religion in humanitarian contexts, particularly those outlined by Tadros (2020), which emphasize the need for context-sensitive proxies when direct data is unavailable. Using proxy data developed by the Observatory for Religious Freedom in Africa (ORFA) and particularly focusing on state-level ratios of religiously motivated violence, we construct a reasoned estimate of Christian displacement.

The remainder of this paper is organized as follows. Section 2 explores the limitations of official displacement datasets, highlighting institutional and methodological reasons for the absence of religion-specific data. Section 3 presents the rationale for estimating religious displacement, drawing on qualitative evidence to support the hypothesis that Christians are disproportionately affected. Section 4 reviews alternative methodological frameworks and explains the rationale for selecting a proxy-based approach. In Section 5, we introduce our findings and describe how data on civilian fatalities from religiously motivated community raids can be used as a proxy to estimate Christian displacement. Finally, Section 6 addresses the ethical and analytical limitations of this model and reflects on the broader implications for understanding religious persecution in Nigeria's displacement crisis.

## 2 Missing numbers: limitations in official data collection

Humanitarian organizations like IDMC, IOM, and UNHCR maintain comprehensive datasets on internal displacement. Yet none disaggregate by religious affiliation. The omission is typically justified by claims that collecting such sensitive data could compromise operational neutrality, trigger politicization, or endanger displaced individuals (see Hurd, 2015).

However, especially in contexts like Nigeria where religious identity is deeply entangled with conflict, this omission risks obscuring critical dimensions of displacement. Scholars and advocates argue that reluctance to include religious data may stem from a broader unease with acknowledging religious persecution as a central dynamic of crises (Tadros, 2020; Coalition for Religious Equality and Inclusive Development (CREID), 2022). While the notion of institutional bias remains conjectural, its potential presence cannot be dismissed.

Another barrier is the allocation of limited resources: due to budgetary and logistical constraints, humanitarian agencies often prioritize immediate life-saving needs such as food, water, and shelter over the collection of granular identity data, including religious affiliation (MacLeod, 2022). Moreover, accurately determining religious affiliation in displacement contexts is inherently challenging. Individuals may mask affiliation from fear, and identities often overlap with ethnicity or regional identity. Armed Conflict Location & Event Data Project (ACLED) researchers confirm this, explaining that assigning religious identity to displaced individuals or events is extremely difficult unless explicitly stated in source material, leading to the exclusion of such details from their coding (Armed Conflict Location and Event Data Project (ACLED), 2022).

In turn, this results in datasets that underrepresent the religious dynamics of violence and displacement sacrificing contextual nuance in favor of conservative coding. In many cases, data exclusions are not due to resources but stem from methodological caution (Armed Conflict Location and Event Data Project (ACLED), 2022).

Additionally, broad structural issues compound the problem. As noted in displacement data innovation reviews, limited infrastructure, weak statistical capacity, and political sensitivities particularly around religious demography hamper more detailed data collection in fragile settings (MacLeod, 2022). Concerns about misuse such as surveillance or selective targeting of religious minorities also dissuade agencies from collecting religious data (MacLeod, 2022).

Ultimately, the absence of religious disaggregation in displacement tracking significantly undermines comprehensive analysis, especially in multi-faith societies experiencing communal violence. To better understand and address displacement drivers, religious variables must be reintegrated into data frameworks.

## 3 Rationale for estimating religious displacement

Despite institutional constraints (or reluctance) to collect or publish religiously disaggregated displacement data, there is a growing body of evidence that justifies estimating the proportion of Christians among Nigeria's IDPs. Numerous field reports and independent studies such as those published by ODI, ORFA, and IIRF document the systematic targeting of Christian communities, particularly in regions afflicted by Islamist insurgencies and communal violence (Observatory of Religious Freedom in Africa, 2024; Open Doors International, 2025; International Institute for Religious Freedom, 2024; Petri and Bainbridge, 2024).

The report *No Road Home* by International Institute for Religious Freedom (2024) presents a detailed analysis of Christian displacement in states like Benue and Plateau. It highlights deliberate and repeated attacks on Christian villages, churches, and faith leaders, as well as the abandonment of entire Christian farming communities due to the threat of violence. Similarly, Open Doors' annual *World Watch List* identifies Nigeria as one of the most dangerous countries in the world for Christians, citing religiously motivated killings, abductions, and displacement (Open Doors International, 2025).

Armed Fulani Herdsmen, among other perpetrators, have been particularly brutal in their campaign against Christians, frequently targeting churches, Christian schools, and communities (Observatory of Religious Freedom in Africa, 2024). These groups have openly declared Christians to be enemies of their ideology, and their attacks have forced tens of thousands to flee, often without formal documentation or humanitarian assistance (Open Doors International, 2025).

While local and national government strategies increasingly acknowledge the role of civil society and community institutions in responding to displacement, explicit attention to religious targeting remains limited. For instance, Borno State's 2025–2027 durable solutions strategy recognizes the role of religious institutions in peacebuilding and reintegration but does not specifically address the religious motives underlying many displacement events (Government of Borno State, 2024). The plan emphasizes the importance of inclusive approaches that respect cultural and religious diversity, but it largely overlooks the scale and pattern of targeted violence against Christian communities.

In the absence of formal disaggregation, proxy estimation becomes essential. Religious targeting is not incidental in Nigeria's conflict landscape; it is a core dimension of many displacement events. Estimating the religious composition of the displaced population is therefore not just methodologically feasible; it is ethically necessary for understanding the true nature of Nigeria's internal displacement crisis and ensuring that humanitarian responses are culturally and religiously sensitive.

## 4 Methodological approaches

This study explores various strategies for estimating religious displacement in the absence of direct religious identifiers. Taking on the challenge formulated by Douglas W. Hubbard in *How to Measure Anything: finding the Value of “Intangibles” in Business* (2014), it should in fact be possible to devise a method for estimating the religious affiliation of IDPs in Nigeria (an “intangible”) even with limited data. As Hubbard argues, the first step in any measurement effort is to gather what is already known: one typically has more useful information than initially believed and often needs less data than assumed (Hubbard, 2014).

In this context, event-level data collected by ORFA includes significant information on the religious identity of victims of violence, particularly killings and abductions, which may serve as an indirect proxy for estimating the religious composition of displaced populations. The International Institute for Religious Freedom (IIRF), drawing on ORFA and other sources, has demonstrated that Christians in Nigeria are disproportionately affected by violence relative to their population size (Petri and Bainbridge, 2024). These findings form the statistical foundation for our approach, which assumes that religious bias in killings data may mirror bias in conflict-related displacement.

**Figure 1:**
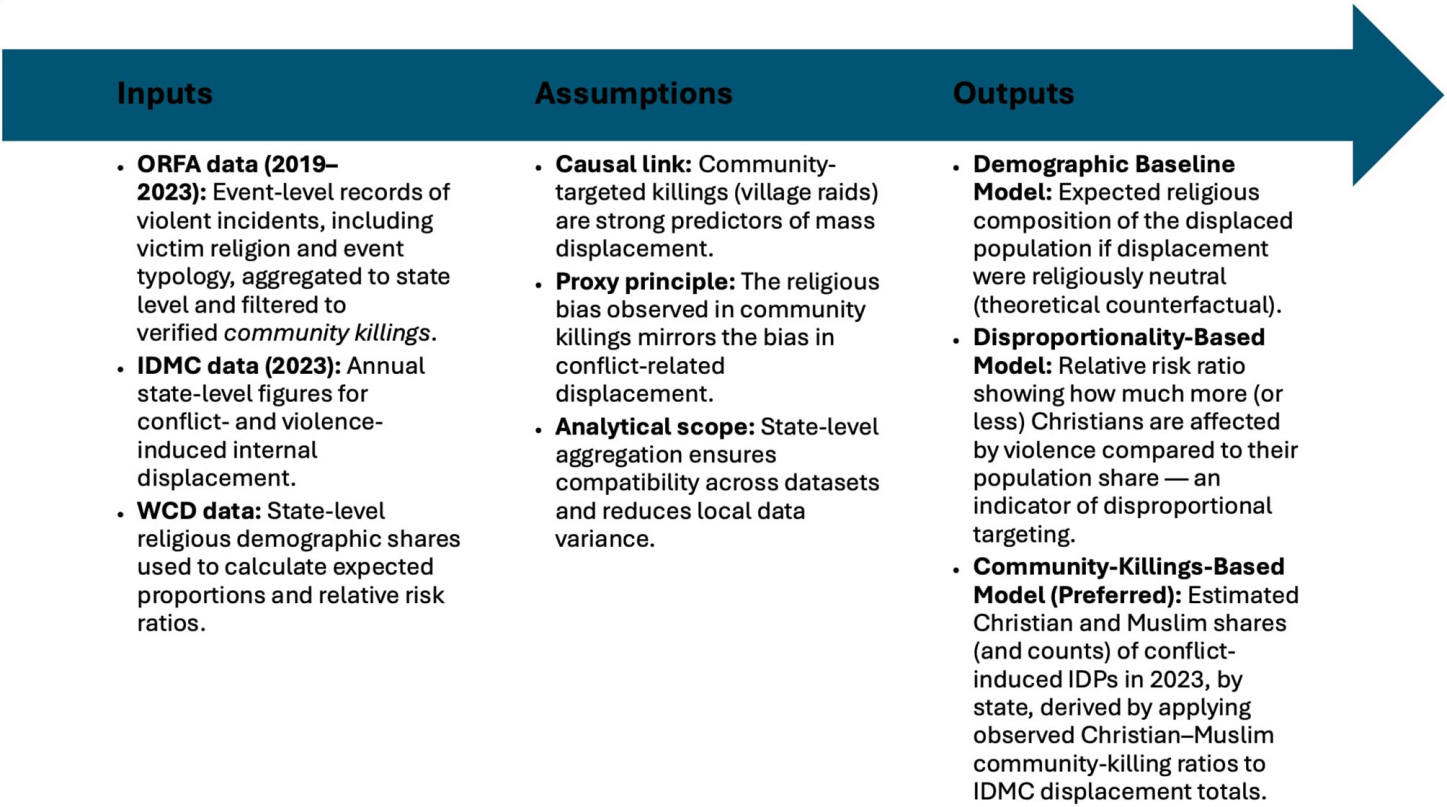
Inputs, assumptions, and outputs of the proxy-based estimation model for religious displacement in Nigeria.

A key strength of the ORFA dataset is its categorization of violent events, distinguishing between “community killings” (targeting entire communities, often in village raids) and other forms of violence, such as intra-group rivalries or attacks on public officials. This distinction is crucial for our purposes, as community raids can be assumed to be more likely to result in mass displacement, as ORFA researchers report. If the higher rate of such attacks on Christian communities correlates with forced migration, then observed ratios of religiously identified killings provide a viable basis for proxy estimation. To clarify the inferential logic of this approach, Figure 1 summarizes the proxy model linking violence data to displacement estimation.

This assumption is supported by field accounts. For example, a case study from southern Kaduna documented by Truth Nigeria describes a series of raids on Christian farming villages over a 35-day period in which residents were either killed or forced to flee, often permanently (Truth Nigeria, 2025). Such events reflect the kind of indiscriminate violence captured in ORFA's “community killings” category. This kind of violence is not only lethal but deliberately dislocating, with clear religious or ethnic motives.

Available displacement-reports document both patterns of lethal violence and corresponding forced migration, partially confirming this assumption. For example, the report *No Road Home* (International Institute for Religious Freedom, 2024) highlights that Nigeria is host to approximately 3.4 million internally displaced persons (IDPs) as of 2023, and that in northeastern states such as Borno State and Middle Belt states such as Plateau State, extremist violence directed at Christian-majority rural communities has directly precipitated large displacement flows. According to interviews conducted in Borno and Plateau, many Christians reported mass raids, destruction of churches and farmlands, and entire villages fleeing in the immediate aftermath of attacks. While the report does not present a one-to-one ratio connecting killings to displacement, these qualitative findings suggest that spikes in community-targeted violence coincide closely with sharp increases in displacement from affected areas. Together, these data points reinforce the plausibility of using “community killings” as a proxy indicator for religiously structured displacement, albeit with caution given the absence of systematically disaggregated displacement data.

The IDMC identifies two primary causes of internal displacement in Nigeria: natural disasters (especially floods) and conflict or violence. This study focuses exclusively on displacement caused by conflict and violence, as natural disasters generally lack a religious dimension. Nigeria's dual burden of displacement highlights the need for accurate, context-sensitive analytical tools, especially when states are expected to formulate comprehensive response strategies.

Borno State's 2025–2027 strategy for durable solutions to internal displacement exemplifies such efforts. It acknowledges the potential role of religious institutions in peacebuilding and reintegration (Government of Borno State, 2024). Although not always named explicitly, faith-based organizations are likely considered part of the broader civil society framework. Yet, despite recognizing cultural and religious diversity, the strategy fails to address religiously targeted displacement, a silence that underscores the need for alternative data-driven models to assess this overlooked dimension.

Our analysis operates at the state level, the lowest feasible geographic unit given current data limitations. Although Local Government Authority (LGA) data could provide greater precision, it is inconsistently reported and lacks the reliability needed for robust modeling. Moreover, both ORFA and the World Christian Database (WCD) provide data only at the state level, justifying our decision to maintain geographic consistency (Johnson and Zurlo, 2024).

We recognize, however, that significant intra-state variation likely exists, as patterns of religious violence and displacement can be highly localized within specific LGAs or clusters of villages. A finer-grained analysis could, in principle, reveal important sub-state disparities, but such an exercise would require more granular and systematically reported data than are currently available. Nonetheless, at a broader scale, the spatial distribution of community raids shows recurrent clustering within states or regional zones rather than random dispersion. Community raids typically do not recur in the same localities year after year but tend to concentrate within these wider areas. Aggregating data at the state level thus helps stabilize year-to-year variation and supports more reliable inter-state comparisons.

Temporally, the study focuses on annual displacement flows—specifically, the number of people displaced by conflict or violence in 2023—rather than the cumulative stock of IDPs in each state. This decision reflects the intent to match incidents of violence with contemporaneous displacement, enhancing the relevance and accuracy of our model. Preliminary inspection of the ORFA data for 2019–2023 suggests that, despite fluctuations in absolute numbers, the relative ratios of religiously targeted violence display a high degree of temporal consistency, indicating that the proxy relationship between community killings and displacement is stable across multiple years rather than limited to a single observation period.

By integrating ORFA's religiously disaggregated violence data with IDMC's conflict-related displacement figures, this study aims to construct a robust, ethically responsible, and methodologically transparent model for estimating Christian displacement in Nigeria. The following sections present three estimation strategies and explain the rationale for selecting the preferred approach.

### 4.1 Analytical framework and working hypotheses

This study discusses the plausibility of three estimation strategies for assessing the religious composition of Nigeria's internally displaced population in the absence of direct religious identifiers. It operates under three working hypotheses:

- H1 (Baseline): Displacement is religiously neutral and reflects state-level population shares.
- H2 (Disproportionality): Displacement risk varies proportionally with the relative risk of being killed due to religion.
- H3 (Community Displacement): The religious composition of community-targeted killings approximates the composition of displacement caused by conflict and violence.

Each hypothesis corresponds to one of the estimation models described below. The models are evaluated for internal consistency, empirical plausibility, and parsimony of assumptions.

### 4.2 Data sources

Two open and independently verified datasets were used in this study. The first is ORFA, which provides event-level data on violent incidents, including the religious identity of victims and the typology of each event, aggregated to the state level for the years 2019–2023. The second is IDMC dataset, which reports annual state-level figures for conflict- and violence-induced displacement for 2023. ORFA events were filtered to include only verified cases of killings associated with conflict or community attacks.

### 4.3 Estimation models

Accordingly, the study compares three models: a demographic baseline estimate, a disproportionality-based ratio, and a community-killings-based proxy. Each offers a different lens through which to interpret the religious dimensions of Nigeria's displacement crisis.

#### 4.3.1 Demographic baseline estimate

The first strategy considered in this study is a Demographic Baseline Estimate, which assumes that violence and resulting displacement occur independently of religious affiliation. Under this model, the expected religious composition of the displaced population is proportional to the existing religious demographics of each state. State-level religious breakdowns are drawn from the World Christian Database (WCD), which offers detailed estimates of religious affiliation by region (Johnson and Zurlo, 2024).

For example, in Kaduna State, the WCD estimates the population as 35% Christian, 50% Muslim, and 15% adherents of other or indigenous religions. If violence and displacement were truly random with respect to religion (religiously indiscriminate) then the expected ratio of Christian to Muslim IDPs in Kaduna would mirror the population ratio, yielding an expected displacement ratio of 0.7:1 (35% ÷ 50%).

This model serves as a theoretical baseline. It establishes a reference point against which real-world data can be compared. Deviations from this expected ratio can then be interpreted either as anomalies or, more likely, as evidence of religiously targeted violence. Such a baseline is useful in isolating the influence of religious identity from other potential explanatory variables like geography, class, or ethnicity.

However, while this model offers a neutral benchmark, it is ultimately rejected as a viable explanatory tool in the Nigerian context due to the weight of empirical evidence showing disproportionate targeting of Christian communities. In particular, a recent report by ORFA presents comprehensive statistical evidence that Christians in Nigeria are systematically more likely to be victims of violence than Muslims, even in states where they represent a minority (Petri and Bainbridge, 2024).

This conclusion is reinforced by IDMC's own country-level analyses, which indicate that violence is not distributed evenly across communities but is shaped by local power dynamics and inter-group conflict histories (Internal Displacement Monitoring Centre (IDMC), 2024). The patterns documented by ORFA such as the targeting of Christian villages in Benue and Plateau for community raids are not easily explained by neutral demographic distribution. They suggest a form of conflict that is deeply structured by religious identity.

As a result, while the demographic model offers a useful theoretical counterfactual, it is methodologically inappropriate for capturing the real drivers of displacement in Nigeria. It is set aside in favor of models that directly account for the religious identity of victims and the demonstrated bias in patterns of violence.

#### 4.3.2 Disproportionality-based “estimate”

The second strategy explored in this study is the Disproportionality-Based “Estimate”, which seeks to quantify the relative risk of displacement for different religious groups, rather than determine absolute numbers. This approach compares observed patterns of religiously disaggregated killings with expected outcomes based on population size, thereby revealing whether certain groups, especially Christians, are disproportionately targeted by violence likely to result in displacement.

The key assumption underpinning this model is that killing ratios can serve as a reliable indicator of differential risk. If one religious group is consistently overrepresented among the victims of violence, this implies a higher relative likelihood of that group also being displaced, even if raw displacement figures are unavailable.


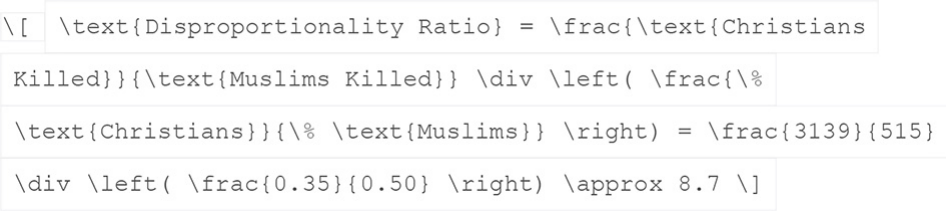


In the case of Kaduna State, for example, ORFA data from 2019 to 2023 reports 3,139 Christians and 515 Muslims killed in violent incidents, resulting in a killing ratio of 6.1:1 (Observatory of Religious Freedom in Africa, 2024). This observed ratio is then compared against the state's religious demographic baseline, which is approximately 35% Christian and 50% Muslim, yielding a baseline ratio of 0.7:1. By dividing the observed ratio (6.1) by the expected ratio (0.7), we calculate a relative risk factor of 8.7. This means that, proportionally, Christians in Kaduna were 8.7 times more likely to be killed than Muslims during this 5-year period, assuming equal population sizes.

While this method does not produce real-world estimates of displacement, it is highly valuable for communicative purposes. It illustrates the extent to which religious targeting skews the dynamics of violence and, by implication, displacement. This kind of proportionality analysis allows policymakers, researchers, and human rights organizations to understand not just how many people are affected, but who is most vulnerable and why.

However, this strategy is not appropriate for predictive or absolute estimation. It does not account for the actual number of displaced persons, nor does it establish a direct connection between killings and individual displacement events. Instead, it offers a risk multiplier that underscores the unequal burden borne by Christian communities in conflict-affected regions.

This strategy is useful in advocacy settings, where demonstrating disproportional risk can help justify targeted protection efforts or specialized humanitarian responses for religious minorities. Nevertheless, for the purpose of estimating real-world displacement figures this method is insufficient on its own. It must therefore be complemented by models that use observed data to approximate actual displacement outcomes.

#### 4.3.3 Community-Killings-Based Estimate

The Community-Killings Model (CKM) estimates the number of displaced persons of each religion in state *i* as:


$$\begin{array}{l} D_{i,r}=T_{i}\times \frac{K_{i,r}}{K_{i,C}+K_{i,M}} \end{array}$$

where *D*_*i, r*_ is the estimated displacement for religion *r* in state *i*, *T*_*i*_ is total conflict-related displacement, and *K*_*i, r*_ represents killings of religion *r* in community-targeted violence.

This preferred strategy for estimating the number of internally displaced Christians in Nigeria is the Community-Killings-Based Estimate. This approach is built on the assumption that community-targeted killings (resulting from violent raids on villages) are a reliable proxy for displacement, as these attacks frequently result in the forced evacuation of entire communities. In contrast, killings of individuals tied to factional disputes, government officials, or other targeted violence are less likely to result in mass displacement.

For the explicit purpose of estimating real-world displacement, this method uses the observed ratios of religiously disaggregated community killings to infer the likely religious composition of those displaced in a given state. Unlike the Disproportionality-Based Estimate (Section 4.3.2), this method does not adjust for population size. Instead, it assumes that if Christians constitute the overwhelming majority of victims in community-targeted killings, they are also likely to make up the majority of those displaced. This approach prioritizes impact over proportionality, aligning more closely with the practical needs of humanitarian response.

In Kaduna, for instance, ORFA data from 2019 to 2023 reports 3,139 Christians and 515 Muslims killed in community attacks, producing a killing ratio of 6.1:1. IDMC data shows that 8,720 persons were displaced by conflict and violence in Kaduna in 2023. Applying the observed 6.1:1 killing ratio directly to the displacement figure yields an estimate that approximately 7,567 of the displaced were Christians, and 1,153 were Muslims.

This model is then replicated across 14 Nigerian states for which IDMC provides disaggregated 2023 displacement data due to conflict and violence. The same method is used: identify the Christian–Muslim killing ratio from ORFA's community-killings data for each state and apply that ratio to the state's displacement total for the year. This allows for consistent, state-by-state estimation of the religious composition of the displaced population without introducing speculative demographic adjustments.

The strength of this approach lies in its simplicity and grounding in observed events. It offers a pragmatic, evidence-based tool for generating plausible estimates in environments where religious identifiers are systematically absent from official displacement data. Importantly, this method aligns with Hubbard's principle of estimating the “intangibles” by leveraging the best available proxies (Hubbard, 2014), making it a methodologically transparent and ethically responsible choice for addressing religious persecution in the context of Nigeria's displacement crisis.

### 4.4 Limitations and validation

This exploratory estimation framework is designed for heuristic and comparative use rather than precise prediction. The lack of religious identifiers in displacement data precludes direct validation, but the model's assumptions are triangulated with independent field reports and consistent spatial patterns of religiously targeted violence. Future studies could enhance robustness through sensitivity analysis or Bayesian updating as more granular data become available.

## 5 Findings and proxy estimation

Using ORFA's state-level community killing ratios as a proxy, we applied the preferred estimation model to displacement data reported by IDMC for 2023. This method enables a preliminary approximation of the number of Christians displaced by violence in the absence of official religious disaggregation. One advantage of this approach is that it remains grounded in event-based evidence; only the religious composition is estimated, based on documented patterns of community-targeted killings.

Because the underlying data and estimation logic are consistent across states, all reported figures should be interpreted as approximate indicators rather than exact counts. To avoid suggesting unwarranted precision, values have been rounded to the nearest multiple of hundred. The overall model confidence is classified as “medium,” reflecting the reliability of ORFA and IDMC data but recognizing the inferential nature of the proxy relationship.

We implemented both the population-based (Model 1) and the killings-based (Model 3) approaches, allowing for a direct quantitative comparison of their outcomes. Applying these two methods to the data supplied by IDMC for 2023 provides the following estimations at Nigerian State level (Table 1):

**Table 1:** Proxy-based estimates of christian displacement from violent incidents in Nigeria, 2023 (rounded values).

**State**	**Violent displacements 2023**	**Christians—population-based estimate (model 1)**	**Christians—community-killings-based estimate (model 3)**
Kaduna	8,700	3,100	6,100
Kano	200	0	0
Katsina	44,600	3,100	10,900
Sokoto	17,200	900	3,300
Zamfara	44,400	2,200	6,400
Benue	29,900	22,400	25,300
Nasarawa	300	100	200
Plateau	25,500	15,300	19,700
Adamawa	1,200	400	700
Bauchi	1,000	100	200
Borno	76,100	15,200	28,700
Gombe	400	100	300
Taraba	39,200	11,800	29,500
Yobe	2,100	200	400

Figures are rounded to the nearest multiple of 100 and reported only for states with available data. Estimates are derived from proxy-based models using population and community killings indicators. Results should be interpreted as approximate rather than exact counts. Overall model confidence: medium.

This model yields real-world estimates for real-world events. It does not rely on the assumption that Christian and Muslim populations are equal across states (which they are not), and instead maps religious targeting as observed. However, these estimates should be understood as indicative approximations rather than exact measurements, given the constraints of proxy-based inference.

**Figure 2:**
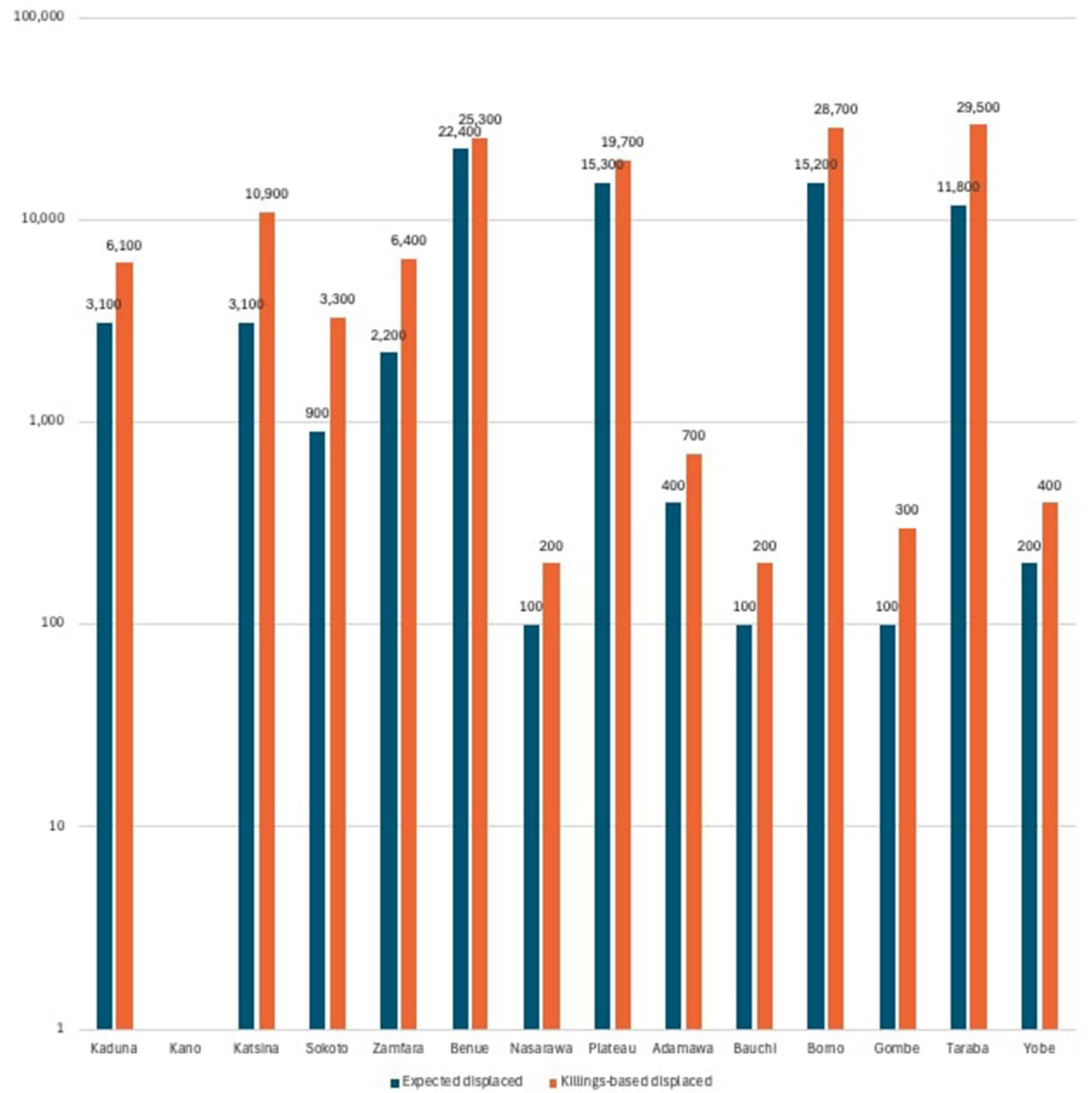
Thousands of Christians displaced per Nigerian State in 2023.

As the table shows, the killings-based proxy consistently produces higher estimates of Christian displacement than the population-based model. Figure 2 presents the comparison using a logarithmic y axis, which makes both low and high displacement values more clearly interpretable across states. Even on the log scale, the chart shows that the killings-based proxy consistently yields higher figures for Christian displacement than the population-based model. This discrepancy underscores the importance of religious targeting in understanding Nigeria's displacement crisis.

The comparison between Models 1 and 3 reveals a consistent and significant disparity in nearly every state analyzed. In 14 Nigerian states, the killings-based method produces higher estimated numbers of displaced Christians than the demographic baseline would suggest. This reinforces the hypothesis that violence and the resulting displacement are not religiously neutral, but instead disproportionately affect Christian communities, particularly in regions characterized by insurgency, communal conflict, and ethno-religious conflict.

This pattern is most evident in states like Taraba, Borno, and Katsina, where the killings-based model nearly doubles or even triples the number of displaced Christians compared to what the population-based estimate suggests. In Benue and Plateau, both known hotspots of religious and communal tension, the already high population-based estimates are substantially exceeded by the killings-based projections, indicating particularly intense targeting of Christian communities. These findings corroborate previous reports from ORFA and the International Institute for Religious Freedom (IIRF), which document widespread attacks on Christian villages and places of worship in these regions.

Regarding Model 2 (the disproportionality model), we did not—and indeed cannot—implement it empirically. As explained in Section 4, this approach serves a purely illustrative purpose: it highlights how religious targeting skews the patterns of violence and reveals the unequal risk borne by Christian communities. However, it cannot generate real-world displacement estimates because it does not quantify actual population movements or link killings to displacement events. Its function is explanatory, not predictive. For that reason, Model 2 is retained as a conceptual reference but excluded from any empirical or hybrid modeling.

Importantly, our results also have implications for humanitarian policy and religious freedom advocacy. If official data collection efforts fail to account for the religious dimension of displacement, then humanitarian aid distribution, peacebuilding strategies, and reintegration programs may miss critical aspects of the conflict. Faith-based communities that have borne a disproportionate share of violence and displacement risk being further marginalized if their unique vulnerabilities are not recognized in state and international responses. This proxy-based model thus serves not only as a methodological innovation, but as a corrective lens for understanding Nigeria's internal displacement crisis.

## 6 Conclusion and limitations

The findings of this study suggest a notable pattern of religious targeting across much of Nigeria, with Christian communities disproportionately affected by displacement linked to community-targeted violence. In proportional terms, the states of Katsina, Sokoto, and Zamfara exhibit the highest levels of disparity between estimated and expected displacement outcomes based on religious demographics. These three northwestern states, which form a geographically contiguous zone, are already recognized in displacement literature as a connected corridor of insecurity (International Organization for Migration (IOM), 2019). Their shared exposure to banditry, insurgency, and intercommunal conflict may partly explain the heightened vulnerability of religious minorities in this region.

In absolute numbers, Borno and Taraba contribute the most to estimated Christian displacement under the community-killings-based model. These findings are consistent with long-established patterns of targeted violence in the northeast and central belt, where attacks on Christian communities have been frequent and often systematic. The high displacement figures from these states reflect both the scale and severity of religiously motivated violence.

Nevertheless, the model presented here has important limitations. First and foremost, it rests on the assumption that a consistent relationship exists between community killings and forced displacement, a relationship that appears likely based on qualitative evidence but has not been empirically verified. While numerous field reports suggest that such violence often results in population flight, this correlation cannot be guaranteed in all cases. Some communities may endure attacks without relocating, while others may flee preemptively in anticipation of violence. Second, the model assumes that religious bias observed in killings is mirrored in displacement patterns; this may not always hold true, particularly in multi-ethnic or politically complex areas. Third, displacement is influenced by multiple, intersecting factors including ethnicity, economic status, geography, and local governance. This makes it difficult to isolate religion as the sole or primary driver.

In addition, the quality and granularity of available data remain challenges. State-level aggregation obscures local variation, and the absence of official religion-disaggregated displacement figures necessitates reliance on proxy indicators. All aggregations and calculations were performed using reproducible scripts, available upon request, to ensure methodological transparency and replicability. The ORFA dataset, while rich, may still underreport certain incidents, and IDMC data may miss smaller or undocumented displacement events.

Most fundamentally, the assumptions underpinning this study, while reasonable given current evidence, may be incorrect. It is entirely possible that violence and displacement do not align as neatly as the model suggests, or that the religious identity of victims and displaced persons does not follow the same pattern. Therefore, findings should be interpreted as indicative, not definitive.

Despite these limitations, this study provides a useful and transparent starting point for estimating religious displacement in contexts where direct data is unavailable. While this study does not include spatial visualization, the proxy-based displacement estimates could readily be represented in a state-level choropleth map to illustrate regional disparities and concentration patterns. Future research could also apply spatial autocorrelation methods (e.g., Moran's I or LISA cluster mapping) to examine potential spatial clustering or gradients between the intensity of religiously motivated violence and estimated Christian displacement across Nigerian states.

In principle, the inferential approach adopted in this study would be unnecessary if displacement datasets systematically recorded the religious affiliation of affected populations. The analysis therefore underscores the need for more comprehensive, disaggregated reporting in humanitarian data systems and calls attention to the overlooked religious dimensions of forced migration in Nigeria. Ultimately, this model contributes to a broader effort to understand and document the role of religious persecution in shaping displacement patterns, informing both academic inquiry and policy intervention.

## Data Availability

Publicly available datasets were analyzed in this study. This data can be found at: https://orfa.africa.
